# Distributed Sensor Architecture for Intelligent Control that Supports Quality of Control and Quality of Service

**DOI:** 10.3390/s150304700

**Published:** 2015-02-25

**Authors:** Jose-Luis Poza-Lujan, Juan-Luis Posadas-Yagüe, José-Enrique Simó-Ten, Raúl Simarro, Ginés Benet

**Affiliations:** University Institute of Control Systems and Industrial Computing (ai2), Polytechnic University of Valencia (UPV), Camino de Vera, Valencia 46022 , Spain; E-Mails: jposadas@ai2.upv.es (J.-L.P.-Y.); jsimo@ai2.upv.es (J.-E.S.-T.); rausifer@ai2.upv.es (R.S.); gbenet@ai2.upv.es (G.B.)

**Keywords:** middleware, distributed architecture, intelligent control, DDS, NCS, quality of service (QoS), quality of control (QoC)

## Abstract

This paper is part of a study of intelligent architectures for distributed control and communications systems. The study focuses on optimizing control systems by evaluating the performance of middleware through quality of service (QoS) parameters and the optimization of control using Quality of Control (QoC) parameters. The main aim of this work is to study, design, develop, and evaluate a distributed control architecture based on the Data-Distribution Service for Real-Time Systems (DDS) communication standard as proposed by the Object Management Group (OMG). As a result of the study, an architecture called Frame-Sensor-Adapter to Control (FSACtrl) has been developed. FSACtrl provides a model to implement an intelligent distributed Event-Based Control (EBC) system with support to measure QoS and QoC parameters. The novelty consists of using, simultaneously, the measured QoS and QoC parameters to make decisions about the control action with a new method called Event Based Quality Integral Cycle. To validate the architecture, the first five Braitenberg vehicles have been implemented using the FSACtrl architecture. The experimental outcomes, demonstrate the convenience of using jointly QoS and QoC parameters in distributed control systems.

## 1. Introduction

Communication requirements are increasing in line with requirements for intelligent distributed control systems. The role of middleware in supporting distributed control systems has also evolved in terms of characteristics. Among the characteristics now required by middleware are time management support and message flow control.

To offer these features, architectures must have formulas that enable monitoring and assessment of these characteristics. These formulas also give rise to the parameters of quality of service (QoS). To manage these parameters, the architecture must provide mechanisms so that they can be configured according to requirements. These mechanisms are known as QoS policies. In addition to evaluating the service provided, the architecture should be able to assess internal compliance with the control objectives, and this aim introduces the concept of control of quality (QoC) parameters.

There are a wide variety of systems that use intelligent distributed control architectures. Of these many environments, we can highlight, among other features, wireless sensor networks (WSN) by its dynamic topology, heterogeneity, scalability and ability to cope with node failures. In the other hand, networked control systems (NCS), due to the control requirements, offer other characteristics, *i.e.*, support to transmit large amounts of information or support to communications time management as the message deadline accomplishment. Both types of systems, WSN and NCS, have different needs but they converge in the wireless sensor and actuator networks (WSAN) [1] or Wireless Networked Control Systems (WNCS) [2] as a paradigm of a system that supports both WSN and CNS highlights. Current trends in distributed intelligent control architectures are orientated towards WSAN or WNCS systems.

Considering the above requirements, the principal theme of this work is a design of a distributed architecture for intelligent control that supports QoS through the measurement of parameters and through QoS management policies. These policies must enable a variation in the characteristics of communication in terms of control requirements, as expressed through the QoS parameters. The developed architecture has been called Frame-Sensor-Adapter to Control (FSACtrl).

To determine the requirements of FSACtrl architecture, we have studied the reviews of key authors about the most important features of distributed architectures for system control. We have designed the FSACtrl architectural elements with these features in mind. The elements that support communications are based on the Object Management Group (OMG) Data-Distribution Service for Real-Time Systems (DDS) standard, while the control elements are based on the Sensor Web Enablement (SWE) standard produced by the Open Geospatial Consortium (OGC).

The DDS model is based on the publication and subscription communication paradigm. In this paper, an extension of a series of elements that enable the management of events during system operation is proposed for that part of the DDS standard known as Data Centric Model Publish Subscribe (DCPS). The SWE model is based on the standardization of control through basic elements that are linked to perform more complex processing. This paper proposes an extension to the control model with the inclusion of DCPS model communication components and support offered for QoC.

A design and control simulation environment has been implemented for validation of the architecture. The environment consists of a visual design application of the architectural elements that enable the execution of control algorithms; as well as a mobile robot simulator that can include various types of sensors, data sources, and obstacles in the environment in which the vehicle navigates.

**Figure 1:**
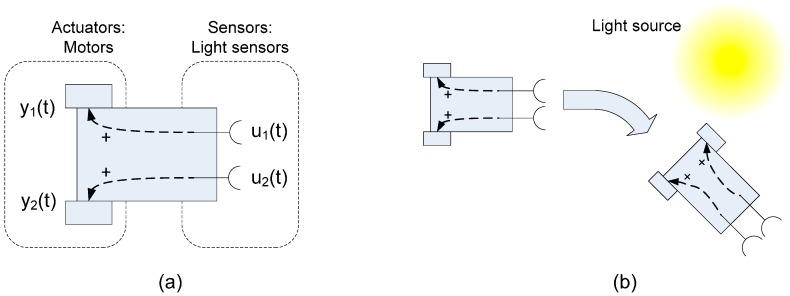
(**a**) Braitenberg vehicle 2 with light sensors and directed wired connection to motors; (**b**) Behaviour resulted in the Braitenberg vehicle 2 when the sensor stimulus (light) appears.

Different Braitenberg vehicles have been implemented as an example of the use of FSACtrl architectural elements. Braitenberg vehicles are widely used in artificial intelligence systems [3]. The main idea of this type of vehicles is connect sensors with actuators and decide the vehicle behaviour in function of these connections. The vehicles are organized in numbers such that lower numbers indicate simple processing, and higher numbers involve complex processing. The Braitenberg vehicles 1, 2 and 3 represent the most basic directed wired sensor-actuator connection [4]. From the Braitenberg vehicle 4, the connections between sensors and actuators are made through processing elements; the behaviour of these vehicles represents more intelligent control systems. Figure 1 shows an example of the Braitenberg vehicle 2.

Braitenberg vehicles are used in a wide range of research fields, from model animal sensory-motor control mechanisms [5] to test the influence of different sensor types in control systems, such as a mobile robot; for example in [6] the Braitenberg vehicles use gas sensors and in [7] the vehicles use pressure sensors.

The first three vehicles lack proper control functions, and thus they are suitable for evaluating the performance of the architecture as middleware. The fourth vehicle includes basic control functions and is used to test the embedding of the control in the communications layer. The fifth vehicle includes logical processing and is used to test how control-based dynamic communication management enables the optimization of system performance.

The results of experimental work developed are satisfactory. We have demonstrated the feasibility of the architecture as middleware for support control, and it has been demonstrated how system performance is optimized by the communications management of part of the control tasks.

The paper is organized as follows: the following section review the related work about the QoS and QoC used in others architectures. Next, the third section presents the design of the FSACtrl architecture by describing its components. Subsections describe its main characteristics about “QoC and QoS Support” and “Events Based Control Support”, respectively. The fourth section describes the environment used to perform tests of the architecture, the implementation prototype and the QoC and QoS parameters that have been considered. The fifth section describes the tests performed and results. Finally, the paper ends with conclusions of experiments done and the future work to be developed.

## 2. Related Work

The optimal control of distributed systems has changed from the systems based on bus-oriented communications to the large industrial systems based on computer networks. The current trend joins all aspects of distributed systems with intelligent control in a concept known as cyber-physical systems [8]. In mobile robot navigation architectures, different components work at different control nodes that are connected through the communication channels.

Systems must be optimum to carry out of the set objectives. To optimize a system it is necessary to have suitable information about which are the features that have more influence on throughput. To manage system performance, the architecture must provide to components all necessary information to build their own quality indicators. One interesting question is, if the quality indicators can be used to take the usual decisions used in the distributed systems, for example moving or cloning a component between two control nodes.

Currently the QoS is used to measure the efficiency of the communications and the quality of component’s services offered [9]. It measures the degree of services compliance, through the QoS parameters [10]. The communications management functions that are oriented to optimize the QoS parameters are known as QoS policies [11].

Among standards to manage distributed communications systems, there are a lot of network protocols, middleware and architectures. The treatment of the QoS is different depending on the standard used. The Common Object Request Broker Architecture (CORBA) defines the QoS by means the concept of messaging policy. CORBA defines 14 policies to cover the basic time, order and routing aspects [12]. The Foundation for Intelligent Physical Agents (FIPA) defines 14 QoS policies mainly in terms of speed and reliability [13]. The DDS standard, proposed by the OMG [14], implements 22 different QoS policies that cover all aspects of communications management: message temporal aspects, data flow and metadata. DDS is based on publish-subscribe paradigm, extended with some elements that connect the application synchronously (readers and writers) and asynchronous (listeners). Therefore, DDS is well suited for implementing distributed intelligent control architectures [15] and is widely used in the professional field [16].

Currently the concept of Quality of Control (QoC) is used to measure the control efficiency [17]. It measures the quality of the control action through equations, generally using the difference between the input signal and the reference signal. Sometimes QoC parameters are used as feedback of control action; thus, the QoC measures the control efficiency and it makes easier the control processing. To measure the QoC, the system must provide separated control nodes that process functionally independent control loops. Among the diverse proposals, the model SWE, by the OGC, monitors and processes sensor data from multiple nodes distributed in space. Besides, SWE includes the organization of the processes.

The control efficiency does not depend exclusively on the algorithms used; the communications efficiency also affects the control action [18], so that there is a relationship between QoS and QoC. To use the different points of view of the QoS with the system’s QoC it is necessary to have a uniform method to obtain the necessary QoS parameters.

As control system complexity grows, it becomes difficult to comply with timing requirements; therefore, the efficiency of the time-driven based control (TBC) approach depends on the context. The Event Based Control (EBC) [19] complements the TBC model by decreasing the messages needed to receive data and send control commands. In the EBC model, messages between sensors, controllers and actuators, are only sent when an important condition is fulfilled. A wide range of conditions can generate events. The most common condition is related to error between the control action and the value obtained, and related to connection maintenance (keep-alive messages). When the EBC model is applied in a NCS, the core of the system is a communications infrastructure based on events with support to distribute efficiently the system information.

There are a great amount of NCS based on events: reference [20] highlights the importance of the network architecture and the protocol used, in addition, [21] emphasizes the importance of measuring the QoC in the NCS. Systems that work in NCS environments must cover these features.

In the EBC model applied to a NCS, the QoC should include the aspects related to the event management, which implies the existence of common parameters with the QoS such as throughput, delay and delay jitter [22]. To provide QoS and QoC support, the FSACtrl architecture is inspired by DDS and SWE models. FSACtrl components arise from the viewpoint of agents. This is because the components, besides offering services, make their own decisions based on the QoS and QoC parameters. Usually, the optimizations of distributed systems are focused on optimizing the communications using QoS to measure the new performance obtained. Simultaneously, the optimization of control systems focuses on optimizing the control algorithms using the QoC to measure the new control efficiency. FSACtrl joins measurement of control and communications performance to obtain a higher performance that using both methods (QoC and QoS) separately.

## 3. FSACtrl Architecture Description

### 3.1. Architecture Components

**Figure 2:**
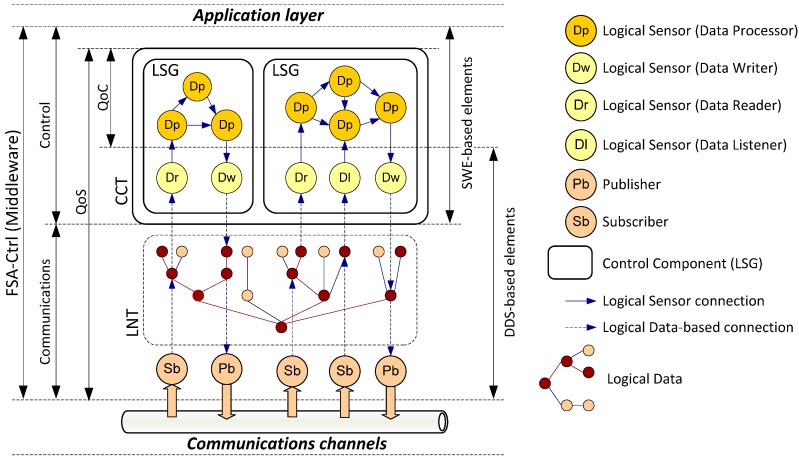
Components of the FSACtrl Architecture.

FSACtrl [23] is a control middleware architecture that allows the distributed control systems design with support to measure QoS and QoC parameters. FSACtrl is organized in two areas: communications and control (Figure 2). The elements that support communications are based on the DDS standard, while the control elements are based on the SWE standard.

The communications area provides a common interface to access communications channels by means of DDS elements: publishers (Pb) and subscribers (Sb). FSACtrl extends the DDS topic with the inclusion of a tree structure called Logical Namespace Tree (LNT). The LNT organizes the information of the system (logical data) in a hierarchical symbolic space.

The control area provides a platform for implementing control algorithms. Basic control elements are known as logical sensors. There are four types: Data Reader (Dr), Data Writer (Dw), Data Listener (Dl) and Data Processor (Dp). Dr, Dw and Dl comply with, respectively, “data reader”, “data writer” and “listener” DDS standard elements. They connect with publishers and subscribers through logical data provided by LNT. Dp complies with the “process model” SWE standard element. Data Processors receive messages from Data Readers, Data Listeners or other Data Processors; they process the messages and send results to Data Writers or other Data Processors.

#### 3.1.1. Data Processors

Data Processors implement simple control algorithms, such as arithmetic additions of messages’ content, or complex control algorithms, such as the path tracking of a mobile robot. The complexity is easily implemented by connecting Data Processors to each other.

**Figure 3:**
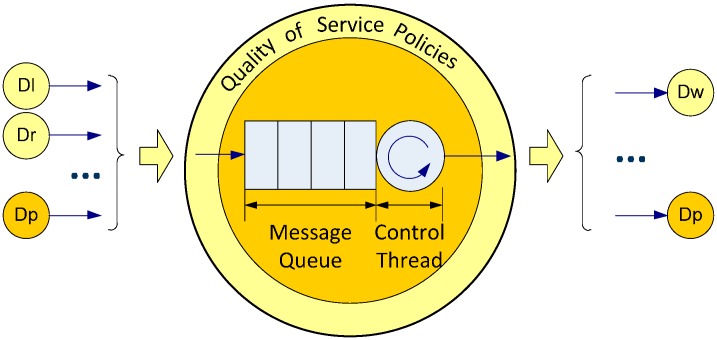
Components of a Data Processor.

All Data Processors have a message queue and a control thread (Figure 3). When a message arrives to a Data Processor it is stored in the queue. Control thread extracts from the queue and processes each message according to its priority. Finally, control thread sends the results.

Message queue parameters (*i.e.*, time between messages, arrival rate, service rate and other performance metrics) provide an overview of the Data Processor performance. These parameters are also known as QoS parameters. Moreover, a QoS policy is a function that dynamically manages communications between elements to obtain specific values for QoS parameters. For example, the deadline QoS policy defines the maximum arrival time of a message. In this case, time between messages will have to be smaller than the time defined in the deadline QoS policy.

QoC parameters depend specifically on the execution context of the control algorithm. QoC parameters are obtained by comparing control algorithm results (theoretical control action) with the measured values obtained after the control action (real control action). For example, in a temperature control system, QoC is obtained from the difference between the theoretic temperature (reference) and the obtained temperature (measured). This difference is called “temperature control error”. If the reference don’t changes during all the control action after the transitory time, the control action is efficient when the temperature control error is closer to zero. In a mobile robot, QoC is more difficult to obtain because there are a great amount of variables that can be necessary to control as robot position, robot orientation or motors speed. Furthermore, in mobile robot navigation, different behaviours have different control algorithms and, and consequently different theoretical control actions with different QoC parameters. Sometimes, two control actions can be contradictory and therefore the architecture must supply to control algorithms some mechanism to decide which is better in a specific navigation context. For example, the “go to” or “keep track” behaviour needs to move the robot towards a goal, but if in the midst of the path there are some obstacles, the “avoid obstacles” behaviour needs to move the robot in the opposite direction.

#### 3.1.2. Control Components

**Figure 4:**
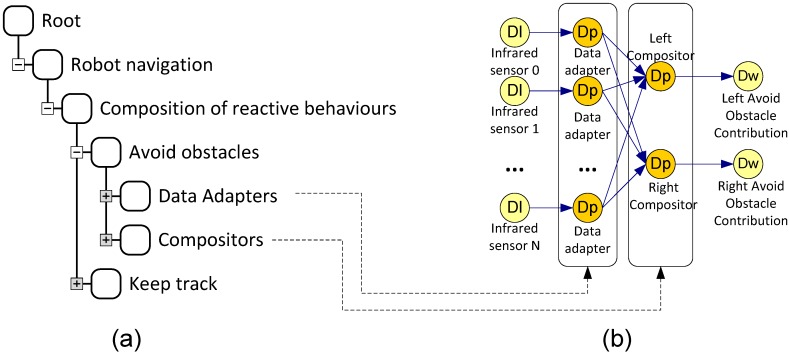
(**a**) Control Component Tree; (**b**) Logical Sensor Graph.

A Control Component or Logical Sensor Graph (LSG) is a grouping of interconnected logical sensors (Figure 4). This component allows the system to implement complex behaviours from simple actions. Control Components can include other Control Components, which creates a tree hierarchy called Control Component Tree (CCT). The root node of the CCT represents the whole system. When a Control Component is close to the root node, it is considered as a deliberative behaviour. Consequently, a Control Component away from the root node is considered as a reactive behaviour.

For example, the Figure 4a shows the behaviours to control a mobile robot by means of a CCT, and the Figure 4b shows implementation details of one of the behaviours by means of a LSG. The CCT shows reactive control components (“avoid obstacles” and “keep track” in the Figure 4). It details how “avoid obstacles” control component consists of “Data Adapters” and “Compositors”.

The LSG shows implementation details of “Data Adapters” and “Compositors”. This LSG contains a Data Listener for each IR sensor, which receive messages from all sensors. “Data Adapters” process the information about the distance of the detected obstacles and alert about the proximity of obstacles. “Compositors” receive the information from all the “Data Adapters” and send control actions to avoid collisions.

#### 3.1.3. Logical Namespace Tree (LNT)

FSACtrl architecture needs organize the information to the control hierarchy. In the DDS model, a topic is a word that represents an information type. This model based on topics is usually implemented in middleware systems [24]. There are many data structures to represent hierarchically the information. For example, Uniform Resource Location (URL) [25] is a good way to identify and organize resources, but it includes extra information as <host> or <port>. Because the DDS middleware layer offers this characteristic to elements, a simple tree data structure can be enough to organize the information. Trees are well-known structures [26] and there are many algorithms to optimize their management, *i.e.*, to obtain subtrees, to modify branches or to locate specifics leafs.

**Figure 5:**
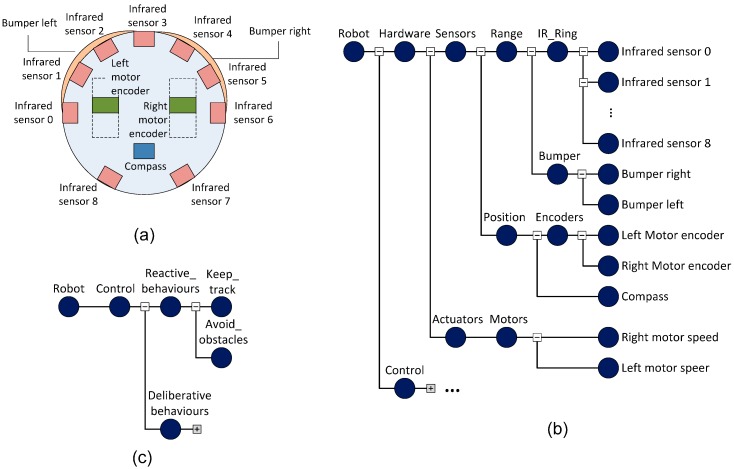
(**a**) Sensors of a robot whose information will be transmitted using a LNT; (**b**) LNT of the mobile robot represented on (a); (**c**) LNT used to organize the control behaviours.

LNT organizes system information by means of items called logical data. A logical data item consists of a sequence of labels separated by the slash character “/”. All logical data form a tree structure that allows elements to select the required information. Figure 5 shows an example of LNT where labels are organized according to the type of sensor or actuator. In this figure, the logical data Root/Hardware/Sensors/Range/IR_ring represents values obtained from all infrared sensors. Secondly, the logical data Root/Hardware/Sensors/Range/IR_ring/IR0_0 represents values obtained only from the IR_0 infrared sensor.

**Figure 6:**
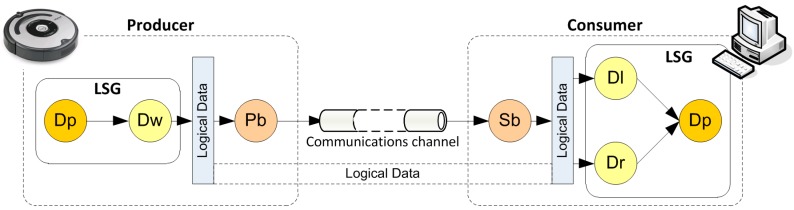
Elements involved in the communication between the robot and control.

Each logical data works as a logical communications channel (Figure 6). When a control component (LSG) produces a value through a Data Processor, this value will be sent to the logical data associated through a Data Writer.

Every control component connected to this logical data, through a Data Listener or Data Reader, will receive the new value. Data Listener or Data Reader sends the new value to Data Processors. A logical communications channel (represented by a Logical Data) is implemented by means of a set of Publishers and Subscribers. LNT connects communications elements with control elements; that is, “Publishers with DataWriters” and “Subscribers with DataReaders or DataListeners”. Every Publisher or Subscriber is linked to a single physical communications channel (*i.e.*, Ethernet, CAN, *etc.*). Pb and Sb convert logical data values to message format required by physical communications channel. So, physical communications channel is transparent to Control Components.

### 3.2. QoC and QoS Support

In a distributed control system, the control action is efficient when it adjusts the difference between the controlled variables and the reference signals to an established value. QoC parameters usually are used to adjust the control action, but can also be used to measure the control action efficiency. In FSACtrl the QoC is used to measure the control efficiency through parameters such as Integrated Absolute Error (IAE) or Integrated in Time Absolute Error (ITAE).

The control efficiency does not depend exclusively on the control algorithms used; it is also affected by the communications efficiency. That is, when QoS parameters values change, QoC parameters values can be affected. So that, to obtain specific QoC parameters values will have to modify QoS parameters values by means of the corresponding QoS policies.

**Figure 7:**
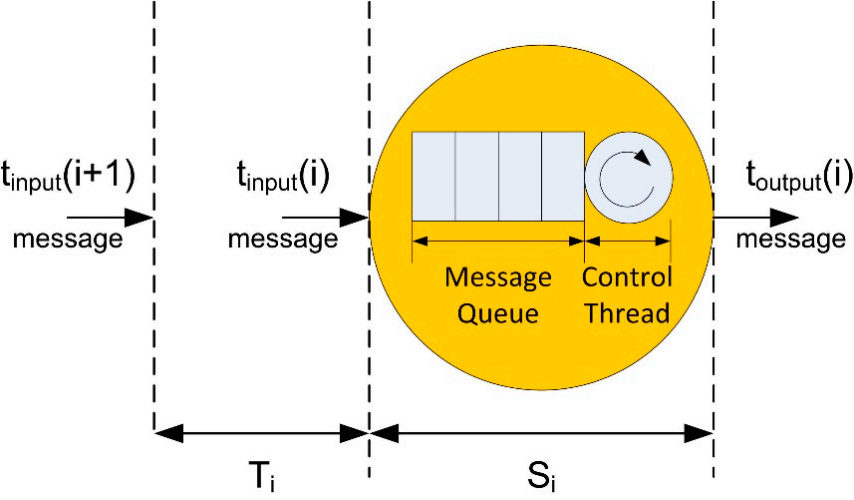
Internal parameters of a basic element.

In FSACtrl, the QoS is based on DCPS model standard and is present in all elements of the architecture. QoS is obtained by means of QoS parameters associated to message queues. As each element has its own message queue, it is possible to define QoS basic parameters through the formulas used in the queuing theory [27] such as service rate, communications load or communications throughput. These basic parameters are shown on (Figure 7) and they are:

- t_input_(i): point in time when the message *i* arrives to the element.
- t_output_(i): point in time when the result of the message *i* leaves the element.
- T_i_: Time frame between consecutive input messages: t_input_(i + 1) − t_input_(i).
- S_i_: Message *i* processing time (include waiting time): t_output_(i) − t_input_(i).

Values of these basic parameters are modified by means of QoS policies defined in DCPS model. Each element will be able to have its own set of QoS policies adapted to its requirements of communication.

Advanced QoS parameters can be obtained from the basic QoS parameters of the elements based on the queue theory as: the element demand (Equation (1)), element service rate (Equation (2)) and element load (Equation (3)):

(1) $${\text{λ}}_{element}=1/E[T_{i}]$$

(2) $${\text{μ}}_{element}=1/E[S_{i}]$$

(3) $${\text{ρ}}_{element}={\text{λ}}_{element}/{\text{μ}}_{element}$$

Based on the element load (Equation (3)) it is possible to obtain more complex QoS parameters and indexes that define the element behaviour; an exhaustive description of the process to transform queue parameters to QoS parameters can be found in [28].

**Figure 8:**
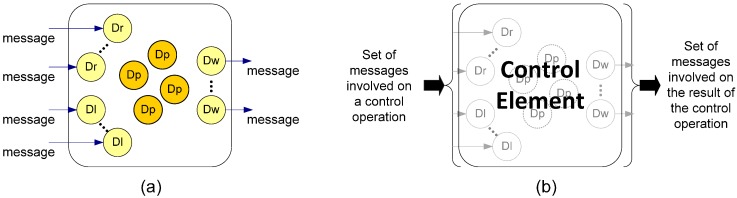
(**a**) Elements involved on the calculation of the internal parameters; (**b**) External parameters considerations.

In the case of control components, the calculation of QoS parameters is more difficult because of the interconnection between logical sensors, so two types of QoS parameters associated to control components have been defined (Figure 8): internal and external parameters.

Internal parameters are calculated by considering QoS parameters of all logical sensors included in the control component (Figure 8a). These parameters provide an overview of the control component operation (*i.e.*, service demand average, maximum service time, *etc.*). Minimum and maximum values of internal parameters allow control elements to detect particular logical sensor problems such as bottlenecks or inefficient configurations.

**Figure 9:**
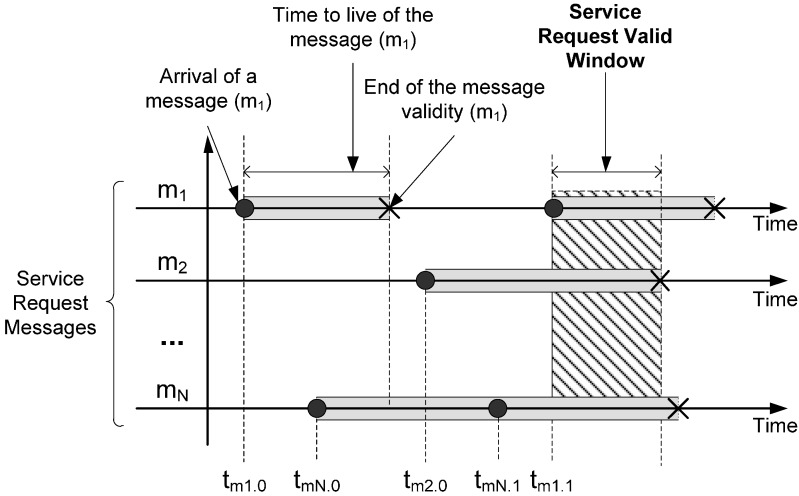
Message parameters used to characterize the internal QoS parameters.

In the other hand, external parameters provide an assessment of the service offered by the component control (Figure 8b). In this case, the calculation of external parameters has to consider the set of messages involved in the control component operation. As these messages can arrive from different sources with different time constraints, the calculation of external parameters will require a multidimensional analysis. This analysis is based on a proposed concept, called Service Request Valid Window (SRVW), which defines the interval time in which the control action can start by using messages with valid information (Figure 9).

**Figure 10:**
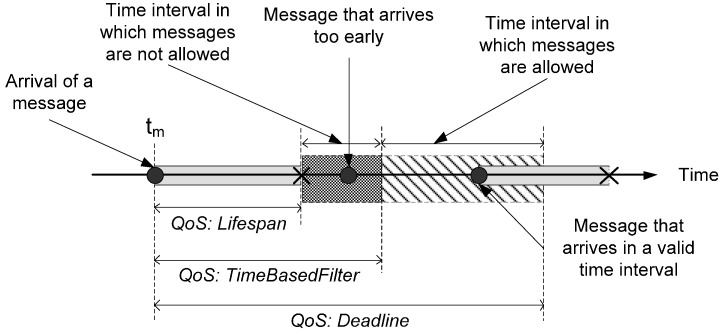
How the three QoS policies (lifespan, timebasedfilter and deadline) define and manage the SRVW for a single message.

For example, a control component that generates control actions to avoid obstacles needs to receive the messages from all infrared sensors in a bounded time. Each message has a lifetime that specifies the interval time during the information of the message is valid, so obstacle avoidance can be guaranteed only if control actions are calculated when all lifetimes overlap. Navigation conditions (such as the robot speed, communications delays or the duration of the calculation of the control action) can change the lifetime of each message. Therefore, conditions change the SRVW boundaries. In order to manage these changes, FSACtrl architecture uses the DDS QoS policies: Lifespan, TimeBasedFilter and Deadline (Figure 10).

The QoS policy Lifespan limits the live time that the control component assigns to the content of the message. The control component uses the TimeBasedFilter policy in order to set the time interval in which incoming messages are not processed. There are some reasons why a message is rejected, for example to synchronize the SRVW or to finish the processing of the previous message. Finally, the Deadline policy is used to limit the time in which a message must arrive. The application of these three policies in every input element makes the SRVW.

Each control component requires different QoS policies values for the same message. The element that sends the message must comply with all the different SRVW requirements of the receivers. When all control components SRVW are synchronized (in terms of QoS compliance), the system works efficiently and complies with the temporal requirements specified by the user.

In the same way as the basic elements, the QoS parameters are obtained based on the time arrival or time processing of a single message, in a control component, the QoS parameters are obtained from the SRVW compliment (all messages involved in the control action arrives into the SRVW). The formulas to measure the QoS are specific to each experiment and robot behaviour, and will be explained in the corresponding experiments section.

#### 3.2.1. Quality of Control in Mobile Robot Navigation

**Figure 11:**
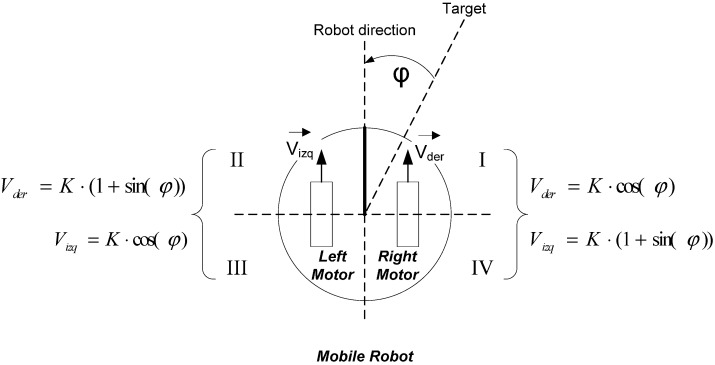
Path error (φ) in a mobile robot used to calculate the QoC parameters.

To obtain the QoC parameters it is necessary to define the control error. In mobile robots, we propose measure the control error by means of the angle that the vehicle deviates from the planned angle in the theoretical analysis of the vehicle mission (Figure 11):

The equations to obtain the QoC parameter can be very different, because of the quality parameter is directly associated to the characteristics of the robot on which it is applied. In the case of vehicle 3.c the QoC is directly calculated with the integral time absolute error parameter ITAE (Equation (4)):

(4) $$ITAE=\int_{t_{0}}^{t_{END}}t\cdot \left|\varphi_{y}(t)-\varphi_{r}(t)\right|dt$$

In the previous equation, φ_y_(t) is the value of the desired angle for a time t, while φ_r_(t) is the real angle obtained in the same instant of time. ITAE parameter considers the navigation error with the same weight during all the navigation time, so that it is very suitable to make global comparisons. The smaller ITAE value, the better quality of navigation of the vehicle is. This is because of the angle obtained from the course is closer than expected angle.

#### 3.2.2. Quality of Service in Mobile Robot Navigation

Middleware manages the QoS. In the case of FSACtrl architecture, QoS is managed by the QoS policies of the DDS standard. In the tests performed the QoS parameters that have been measured are the control component load and the rate of useful messages. The control component load (ρ) is calculated as the rate between the service demand and the service rate of the component (Equation (5)). Due to the architecture elements are made by messages queues, global load is obtained through the pondered rate of each element load (next equation). The K factor is used to balance the most important control components:

(5) $${\text{ρ}}_{global}=\sum_{i=1}^{N}\left(K_{i}\cdot {\text{ρ}}_{i}\right)/N$$

To calculate the load ρ of each component the next equation is used, where λ is the demand for the services requested from the vehicle control and μ is the rate of service provided by the control component. Both of these parameters are expressed in messages per second so that the load is a dimensionless parameter. Closer load to zero better is the control component load:

(6) ρ=λ/μ

The useful messages rate (UM) is obtained using the following equation. The concept of utility of a message can be quite large. In the experimental environment a useful message is considered when the message produces a change in vehicle navigation. The navigation variation is produced when the control action calculated for a measurement is different from the control action calculated for the previous measurement. The closer to one they are, the better the parameter is. The control action in Braitenberg vehicles is performed on the speed of the motors:

(7) $$UM=N_{output(i)\neq output(i-1)}/N_{total}$$

From the two previous equations, the performance (η) of the control can be obtained with the equation shown below. Performance is defined as the satisfactory results obtained in relation to the cost in resources used. The control performance is obtained through the parameters from the Equations (6) and (7):

(8) $$\text{η}=UM\cdot \left(1\;-\;{\text{ρ}}_{global}\right)$$

Through the performance equation, the effectiveness of the control messages related to the resources consumed from the control service can be verified. If the performance value is close to 1, the control action, viewed as a service, is optimized, so that, the value of ITAE indicates how the service improvement affects the vehicle navigation. If the performance value increases, and the ITAE value remains in the same ranges for all cases, the system is optimized without affecting the vehicle navigation.

### 3.3. Events-Based Control Support

There are different methods for tuning a sensor-actuator-based control system, usually based on the PI/PID controller [29]. These models are rules-based, such as first-order plus dead-time, and offer some characteristics like planning support or predictability. However, as described in [30], a Multiple Input Multiple Output (MIMO) system, like the systems to be controlled by FSACtrl architecture, has some problems, especially concerning robustness when is controlled with a rule-based method. To increase the security and the robustness of the PID control, the system events, like deadlines warnings or changes on the external conditions, provide feedback to improve the performance of the control action [31]. Consequently, when a system is characterized by a variation of the operating environment and a dynamic and unpredictable conditions change, the EBC model [32] is recommended. The variety of application fields of the EBC model is extensive, from mobile robots [33] to industrial processes [19].

Currently, the EBC paradigm technology is adopted to implement systems where periodic sampling is not possible (*i.e.*, when no discrete-time model is available) or recommended (*i.e.*, in distributed control systems to improve communications performance decreasing the load of the network) [19].

**Figure 12:**
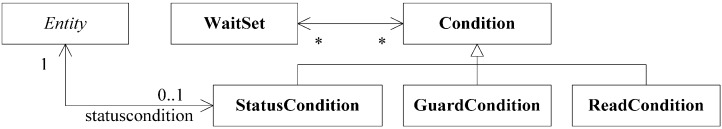
UML class diagram of the DDS elements involved in event management: conditions and WaitSet.

The FSACtrl architecture presents a proposal to extend the event management subsystem of the DDS that offers a simple event management system based on message filtering. Figure 12 shows the main elements of DDS involved on the event management. Entities apply for relevant information by creating one of the types of Condition objects (StatusCondition, GuardCondition or ReadCondition) and attaching it to a WaitSet object, so that, a WaitSet object allows an Entity to wait until one of the attached Condition objects has a “triggervalue” of TRUE or else until the timeout expires.

The aim of the proposal is to improve the event management with three main elements: “Events”, “Conditions” and “Actions”. The proposal is fully compatible with the standard and can be easily added to an existing system.

#### 3.3.1. Components to Supply Event-Based Control

**Figure 13:**
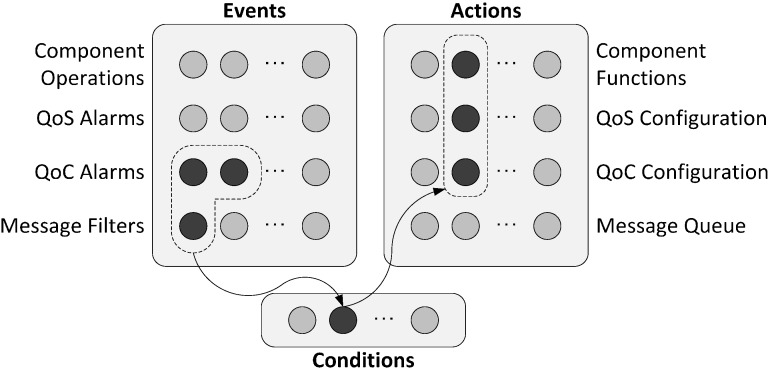
Conceptual model of event management with the source of “Events”, “Conditions” and “Actions”.

Figure 13 shows the proposed event management system with its main components. “Events” are situations that are necessary to be monitored. The “Event” component is similar to the “Condition” DDS element. “Conditions” are group of events using logical operations; the “Condition” component increases the “WaitSet” ability. Finally, “Actions” are the component that implements the effects on the system that are associated with “Conditions”. “Events” are categorized on three types: component operations, quality alarms and message filters.

A component operation happens when a middleware or control component method is called; for example, when a control component starts the control action or when a middleware element is disconnected from a communications channel. Alarms are events associated with the compliance of the quality parameters. In our proposal we include the QoS and QoC parameters. An example of QoS alarm is when a message arrives after the deadline. QoC alarm is related to the content of the message, for example when the internal value exceeds the reference value. Finally, message filters events are triggered when the content of the message is (or not) identical compared to a content pattern; for example, when the message field “source” (user defined) corresponds to a particular control node.

“Conditions” are associations of Events by using basic logic operations (AND, OR). For example, if a message from the node one (message filter) arrives in time (QoS alarm) and the internal value doesn’t exceed the reference value (QoC alarm). The Condition is associated with a DDS element; so that, when the condition occurs, the element knows of its existence. The method to trigger the alarm and to filter the message is the same used on the Remote Network Monitoring protocol (RMON) [34] due to its simplicity and efficiency.

When an element is triggered by a “Condition”, the element can run some function to process the trigger. In our proposal we add a new element called “Action”. “Actions” are associated to a “Condition” and are processed internally in the middleware. Initially, four types of actions have been considered: component functions, QoS and QoC configuration and message queue actions. Component functions are the same functions that can produce events. For example, an action can be disconnecting a middleware element.

The QoS and QoC configurations are the variation of the parameters. For example, in order to increase the temporal limit of messages (QoS configuration) or the control error (QoC configuration).

**Figure 14:**
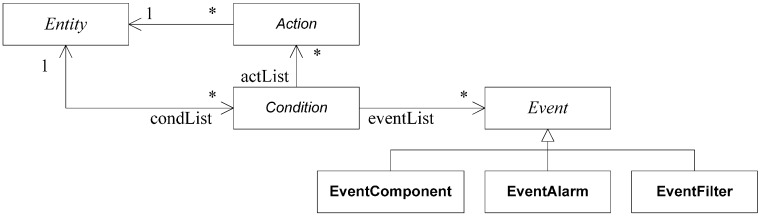
UML class diagram of the proposal elements.

Finally, message queue actions are functions that change the behaviour of the queue, such as priority message, or message removal. So that, “Actions” discharge elements by processing of simple operations. Figure 14 shows the UML class diagram of the proposed elements. In our proposal “Condition” is similar to “WaitSet” DDS element, “Event” is similar to “Condition” and “Action” is the new element.

#### 3.3.2. Event Based Quality Integral Cycle

The control component is optimized by the QoS policies, so that the QoS policies are used to configure the control component parameters. When the adjustment is internal to the control component, QoS parameters are used to change the queue or the control thread behaviour. For example, if the demand rate is increasing, control component can increase the size of the queue.

As already seen in Section 3.2 (QoS and QoC support), the FSACtrl architecture allows message queue parameters to create complex quality indexes. These complex indexes can be used to adjust the system from a global performance point of view. Likewise, it is also possible to include the QoC into the same adjustment process. When QoS and QoC are used to adjust the behaviour of the control component, an integral system quality management is obtained [35].

It is possible obtain a control accomplishment by comparing QoS demanded to control component and QoS offered by the control component. The same process is made for the QoC between the QoC demanded and the QoC obtained. The repetition of the comparing process of the QoS and the QoC is called Quality Integral Cycle.

**Figure 15:**
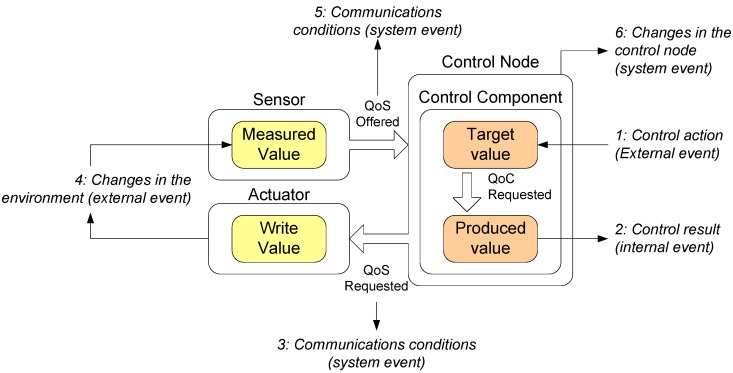
Quality Integral Cycle, QoS and QoC used and events that start the cycle.

The Quality Integral Cycle is useful to a wide range of control functions: to monitor the QoS and QoC of control components (for example to measure the control component load to a determinate algorithm), to evaluate the impact of the behaviour concerned on the control system (for example when a new control component is added in a control node) or to be used in the negotiation communication process (for example to determine the sensors sampling frequency). Figure 15 shows the events that can start a quality integral cycle in a control component.

The events that can start the quality integral cycle can have an external or and an internal origin. External origin events can be of two types: user actions (event 1), for example when the user decides to change the target position of a robot, and environmental changes (event 4), for example when an unknown new obstacle appears in the robot’s path.

Other events are considered internal, and the origin of them is the system as a consequence of a QoS or a QoC event. When the QoC is out of the constrained values (defined by the user), the event 2 starts the quality integral cycle. Same for the QoS, but in this case the event is the third (produced for the outgoing messages) or the 5 event (produced for the incoming messages). Finally, a change in the internal control component conditions (for example, an overload component) produces the 6 event and starts the quality integral cycle.

## 4. Experimental Environment

### 4.1. Control Components Editor

**Figure 16:**
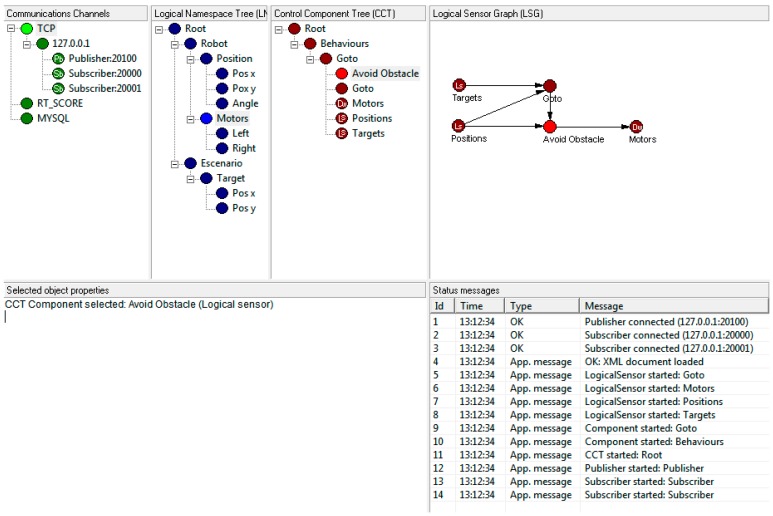
Control components editor.

To facilitate the design and implementation of control algorithms based on the FSACtrl architecture, an editor (Figure 16) that provides a developer graphical environment with predefined component schemas has been developed. FSACtrl architecture elements, such as Logical Sensors, are implemented by using these schemas. The editor launches the control processes and allows inserting, modifying and configuring QoS policies and QoS parameters to each FSACtrl architecture element.

To test the architecture, the control of the first Braitenberg vehicle [36] has been implemented. The interest in Braitenberg vehicles is that the control is based on simple functions that connect different types of sensors and actuators. This provides many messages that allow applications to test the effect of communications configuration in the control efficiency.

### 4.2. Infrastructure to Simulate Mobile Robot Navigation

A mobile robot simulator has been implemented to test the proposed control algorithms. The simulator allows a user to create a 2D environment and insert any number of robots. This environment is composed of a space with different signal sources and rectangular and circular obstacles. All robots used in the study are circular and consist of a simple two wheels controlled by two motors. This configuration allows robots to move in any direction in the simulated environment. For each robot, the simulator has different types of sensors (light, temperature, oxygen, organic, infrared).

**Figure 17:**
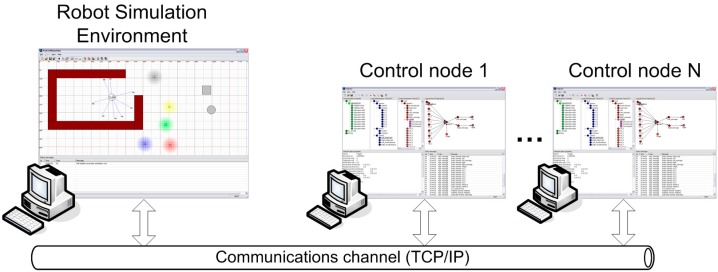
Experimental environment used to test the FSACtrl architecture.

The simulator sends to clients via TCP the data from the sensors of each robot, and it receives, via a TCP server, speeds assigned to each robot motor. Figure 17 shows the topology of the distributed system used to test the architecture. Control nodes are composed of FSACtrl elements developed by using the editor.

### 4.3. Architecture Implementation

Different implementations have been developed and the main aim has been to optimize the resources utilized in the robot navigation. Mobile robots are controlled by a set of control algorithms. Some algorithms, generally algorithms that implement the reactive behaviours, are embedded within the robot and other algorithms, commonly the deliberative algorithms, are implemented in distributed nodes. However, in the case of study, all control algorithms are placed in distributed nodes in order to use a reduced dataset.

**Figure 18:**
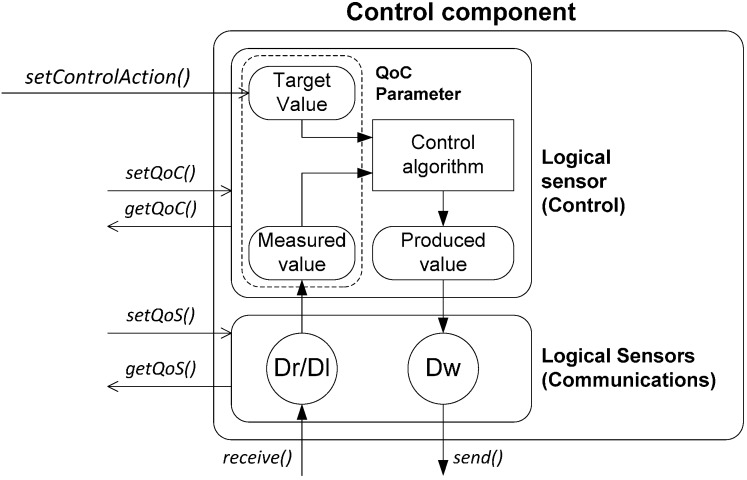
Implementation of the Integral Quality Cycle in a Control Component of the FSACtrl architecture.

Logical control sensors are responsible for providing QoC parameters measured and target values that are part of quality integral cycle. To implement the quality integral cycle, some of the functions of the control components are used and are displayed in Figure 18.

For example, if a user wants to implement the control algorithm for robot navigation behaviour “go to a point” or “follow a path”, the “target value” is the value where the robot should move, usually a position of the robot path. This value is compared to the “Measured Value” to obtain the position error (this error value will be used to obtain the QoC parameters). From the QoC parameter it is possible to obtain a new value to move the robot. If the control algorithm implements the avoid obstacle behaviour the “target value” is the robot security distance according to the current robot speed, the measured value is the distance obtained from the distance sensors and the produced value is the motor speed related to every wheel. These aspects will be detailed in the Experiments and Results section, below.

## 5. Experiments and Results

First of all, the Braitenberg vehicles’ behaviour has been implemented in order to validate FSACtrl architecture. This control algorithm has been executed by using QoS and QoC parameters. The QoS gets to optimize the network load and the QoC gets to optimize the processing load. Secondly, the Braitenberg 5 vehicle has been implemented to validate the EBC support. Finally, with the Braitenberg 5 vehicle, the combination of different behaviours has been validated in order to optimize the communications and control resources used in robot navigation. Following, experiments and obtained results will be described.

### 5.1. Evaluation of QoC and QoS Parameters with the “Go To” Braitenberg-Based Behavior

**Figure 19:**
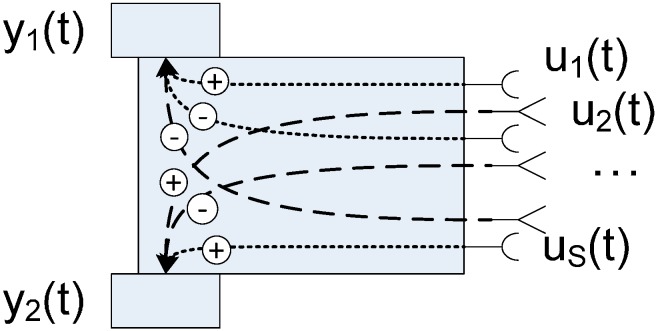
Braitenberg vehicle 3.c with the different input: sensor sources U_x_(t) and the two outputs: motors Y_1_(t) and Y_2_(t).

First three Braitenberg vehicles are based on connections between sensors and actuators without the intermediacy of control processes. The Braitenberg vehicle 3.c (Figure 19) combines the features of previous vehicles, increasing the number of type sensors to four: light, temperature, oxygen and organic.

**Figure 20:**
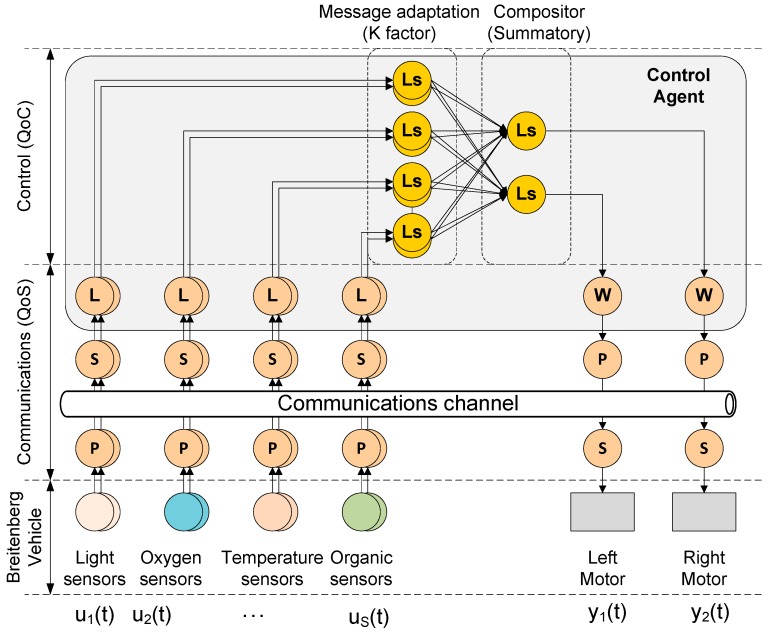
FSACtrl architecture implementation of the Braitenberg 3.c vehicle.

The Braitenberg vehicle 3.c is used to test the FSACtrl architecture as a middleware and how the middleware can be used to optimize the system. FSACtrl elements used to implement the Braitenberg vehicle 3.c are shown in Figure 20.

The Braitenberg vehicle sends sensor values to Subscribers through a specific Publisher for each sensor. Every sensor needs its own QoS parameters (*i.e.*, frequency sampling) and the value of these parameters can change throughout the navigation time. Subscribers send sensor values to the Agent Control Listeners.

The output of each of the composers is calculated from the contribution of each input of the N sensors of the vehicle, weighted by a specific K factor for each sensor. The vehicle calculates the direction that should be taken based on information obtained from the four types of sensors available, using the following equation:

(9) $${Output}_{compositor}=\sum_{i=1}^{N}K_{i}\cdot {Input}_{i}$$

The implementation of the behaviour described on the equation above with an FSACtrl Agent Control needs two steps. The first step performed by the control agent Logical Sensors is the message adaptation by means a weighting factor K. As a result of this step, messages of different sensors have a specific weight to the control action. The second step generates a single control signal to every motor from each sensor input.

#### 5.1.1. Results

Three scenarios on the architecture with the Braitenberg vehicle 3.c have been tested. In the first scenario (scenario one) the control action is obtained without filtered messages optimization and without messages selection optimization: *i.e*., no QoS and no QoC management. In scenario two the control action is obtained with filtered messages optimization and without messages selection optimization: QoS is managed but there is no QoC management. Finally, scenario three obtains the control action with filtered messages optimization and with messages selection optimization: both QoS and QoC are managed.

Message filtering consists of transmitting through the middleware only those messages whose content is different, compared to the preceding message. Message filtering is one of the characteristics specified in the DDS standard recommendations for a middleware. Publishers are the components responsible for this optimization. Message selection produces the improvement of control optimization. This selection is performed by inserting control components that predict changes in the control action. The prediction is made by comparison between messages from different sensors involved in the calculation of control action.

**Figure 21:**
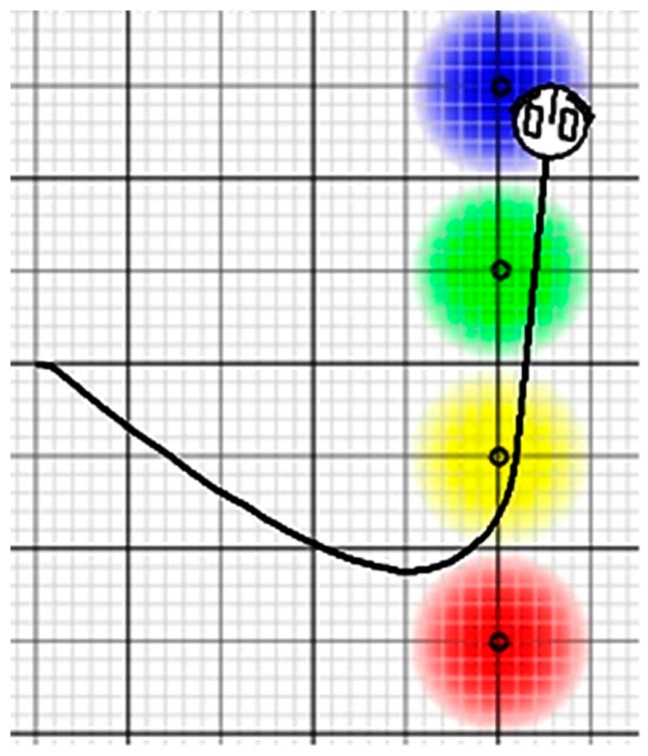
Braitenberg 3.c vehicle follows a path that depends on the location of the sources in the environment.

The environment is a system without obstacles with the four types of sources associated with the four types of sensors of the Braitenberg 3.c vehicle. The vehicle is configured to be attracted by light and organic matter sources, and to be rejected by heat and oxygen sources. The vehicle follows a path that depends on the location of the sources in the environment (Figure 21).

Tests have been performed starting the vehicle in the same position and the sources placed in the same location and changing the middleware according to each described scenario. Table 1 shows experimental values for each of the scenarios described at the beginning of the paragraph. Columns show the average values of the control load, the usefulness of messages rate, the performance of the control element and the value of ITAE. Each row contains the data for each of the scenarios described above.

**Table 1:** Experimental results based on different scenarios (average values).

Scenarios	ρ	UM	η	ITAE
One: Without QoS and QoC management	0.184	0.212	0.173	0.252
Two: With QoS management and without QoC management	0.121	0.323	0.284	0.261
Three: With QoS and QoC management	0.119	0.683	0.602	0.284

Due to the fact the response time of the control service is the same in all scenarios tested; the variation of the control load depends on the message arrival frequency. Because scenarios two and three include a message filtering phase, the control load decreases significantly with respect to scenario one.

**Figure 22:**
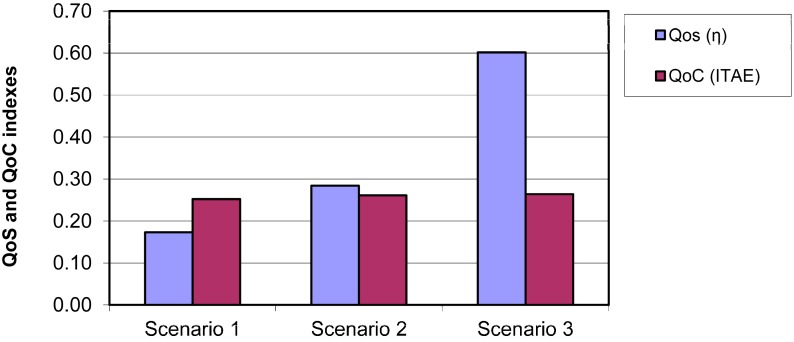
Comparison chart between the η values (QoS) and the ITAE values (QoC).

UM rate changes progressively among the three different scenarios. In scenario two, UM value rises with respect to scenario one because the middleware has filtered some messages that do not generate a control action. However, the most significant improvement of useful message index is produced in scenario three. In scenario three, the control receives only messages that haven’t been filtered in the middleware and in the control prediction. For this reason the message utility rate increases considerably compared with the previous two scenarios. Figure 22 shows the comparison between the service performance indexes (η) and the control indexes (ITAE).

The service performance describes the common contributions of the two QoS parameters and it is a good measure of the performance that the control component provides. The figure shows how performance is directly related to the optimizations used in each scenario. The ITAE parameter is used to check the efficiency of the control service optimizations of the vehicle navigation. In this case, ITAE parameter increases very slightly in relation with the optimized scenario, so that improvements implemented on every scenario scarily affects the quality of the robot navigation.

### 5.2. Evaluation of the Event-Based Control Support with the “Obstacle Avoidance” Braitenberg-Based Behavior

**Figure 23:**
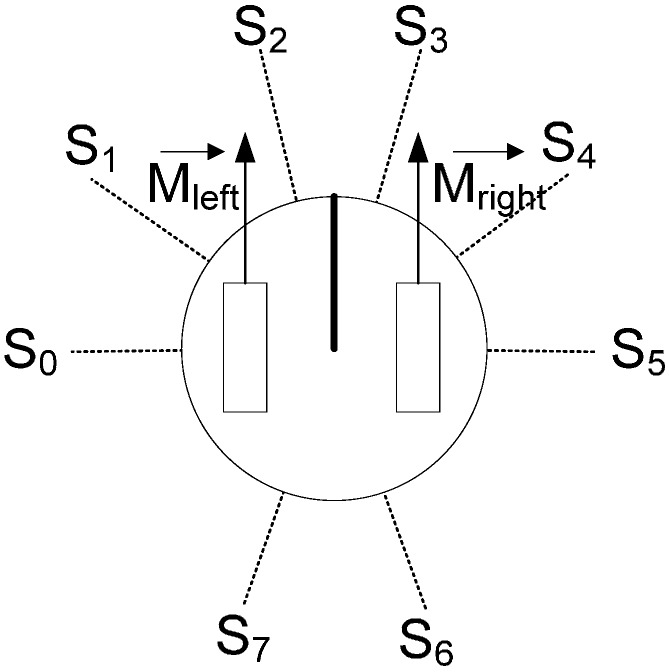
The sensor distribution model used.

In this study the algorithm implemented is the “obstacle avoidance” based on Braitenberg vehicles’ behaviours [36]. This obstacle avoidance algorithm is based on the speed variation of the motor depending on the distance detected by sensors. The robot utilized in the study has eight distance sensors whose distribution (Figure 23) corresponds to a Khepera robot [37], thus the developed algorithms can be compared with existing algorithms.

For each motor, each sensor has a weighting value depending on its position on the robot. The motor speed is obtained combining the sensor weights by means the Equation 10 where *m* is the concrete motor (left or right), *K* is the concrete weight factor applied to sensor *i* and motor m:

(10) $$MotorSpeed(m)=\sum_{i=0}^{7}K_{i}\cdot S_{i}$$

The maximum linear speed of the robot depends on the distance to the nearest obstacle and the sampling period to update motor velocities. The linear speed can be obtained from the Equation (11), where *Sd* is the obstacle distance detected on the current robot path and *T* is the sampling period:

(11) SpeedLimit=Sd/T

**Figure 24:**
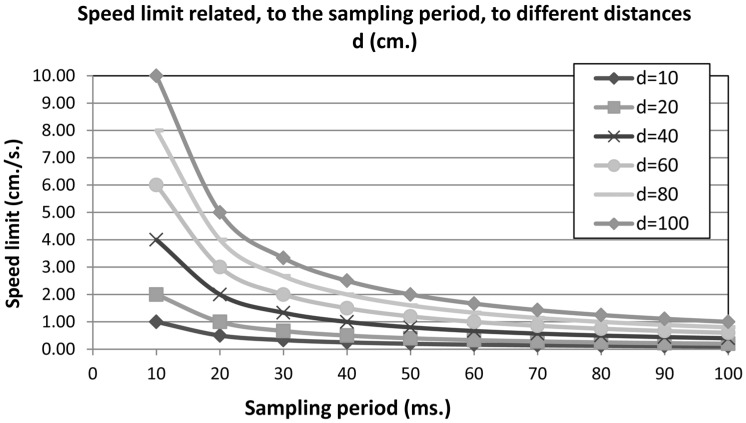
Speed Limit obtained from the distance d and the sample period T.

Figure 24, shows the results of Equation (11) to obtain the speed limit based on the sampling period and different distances. The communication channel defines the maximum sampling period, for example: a sampling period of 10 milliseconds needs a bandwidth to transfer at least 100 messages by second. The frequency of messages sent can be changed through the DDS QoS policies. Therefore, Actions objects can increase or decrease this value automatically without the intervention of control components only with the distance value obtained from the sensors messages. The error in the control of obstacle avoidance behaviour is based on maximizing the distances to obstacles. Therefore, when the robot navigates far from an obstacle, the robot does not need a high sampling rate, so it can decrease the communications load without losing QoC.

#### 5.2.1. Results

**Figure 25:**
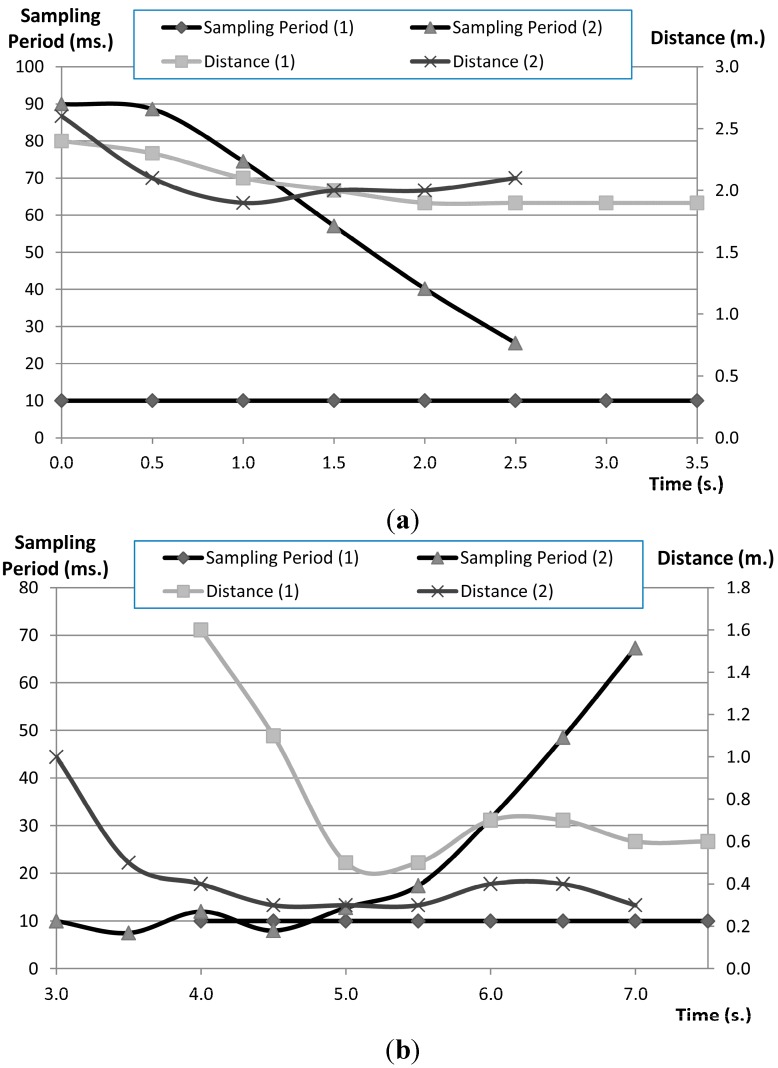
Sampling period and distance variations along time robot navigation, without the proposal (**a**) and using the proposal (**b**) for two different obstacles.

Two scenarios have been tested: robot navigation without event detection in the middleware and the same navigation with event detection and one Action linked by means a Condition. The Event detected in the middleware is the distance obtained from the robot by means a filter. The Condition set involves comparing the distance with the robot speed to obtain the optimal sampling period based on the Equation (11). When middleware doesn’t detect events, the sampling period is set to 10 ms, and motor speed is obtained only with the Equation (10). The experiment measures the sampling period and distance to an obstacle along time. In the case studied the sampling period changes internally in the middleware without the control component intervention. Figure 25 shows the graphs obtained for two obstacles and Table 2 shows the summarized results.

**Table 2:** Experimental results for the two obstacles tested (average values).

Scenarios	Sampling Period (1)	Distance Average (1)	Sampling Period (2)	Distance Average (2)
a. Robot in corridor	10.0	2.1	62.6	2.1
b. Wall in front of the robot	10.0	1.1	9.4	0.6

When the robot navigates through a corridor (scenario a) the average distance is exactly the same for both middleware. This is because the corridor navigation doesn’t involve avoiding an obstacle and the robot can run at maximum speed. However, the absence of obstacles allows navigating with the same efficiency but with fewer messages. Besides, robot reaches the target in less time. In this scenario the proposal does not improve the robot navigation, but reduces the network load. Scenario b is more complex, robot needs avoid an obstacle. As a result, the sampling period is similar. Nevertheless, the robot has more accuracy performing the manoeuvre.

### 5.3. Evaluation of the Communications and Control Resources Optimization with the Composition of Behaviours

In this study, a Braitenberg 5 vehicle which allows composition of behaviours is used. The main issue is to validate the quality integral cycle in navigation behaviours. For this, the 5 vehicle is moved by compositing “obstacle avoidance” and “go to” behaviours with different configurations in the same scenario. First, the 5 vehicle is moved by using fixed sampling periods for communications and control. Second, the vehicle is moved by using dynamic sampling periods in order to optimize communications and control. Sampling periods can be changed by means of the QoS and QoC policy configurations. The parameters used to compare results are: the communications load, the control load and the navigation speed.

#### 5.3.1. Results

**Figure 26:**
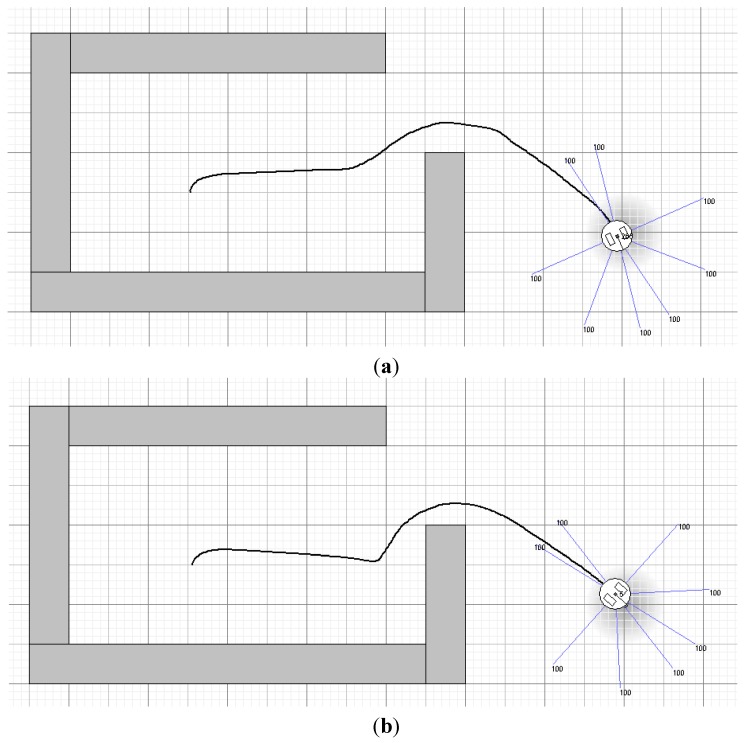
Path I obtained when the navigation when the fixed sampling period is large (**a**) and Path II when the fixed sampling period is short (**b**).

**Figure 27:**
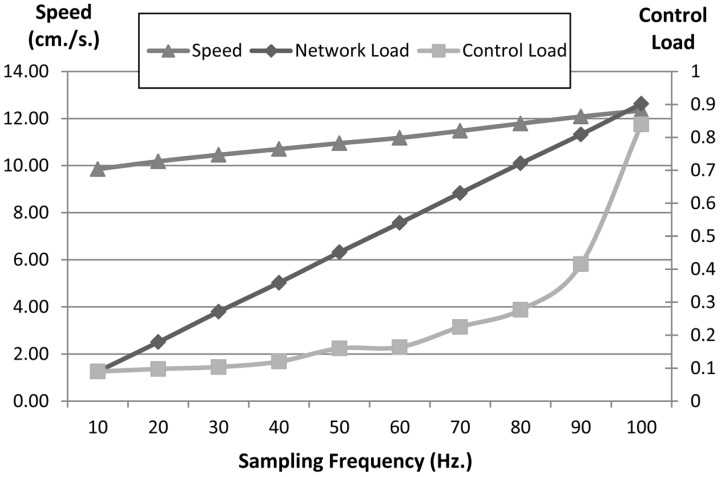
Relation between the sampling rate and the navigation velocity, the communications load and the control load.

The path when the vehicle is moved with a fixed sampling period is shown in Figure 25 and Figure 26. In Figure 25 the fixed sampling period is large, which implies control actions are updated with low frequency and the robot has to move more slowly. On the other hand, in Figure 26 the sampling period is short and the robot can move more quickly. Figure 27 shows the communications load, the control load and the navigation velocity with different sampling periods.

Communications load values are linear with sampling frequency and they are scaled. A communications load equal to 1 means 100 messages per second. On the other hand, a control load equal to 1 means a control component with a 100% load.

Figure 27 shows the relation between the sampling rate and the navigation velocity, the communications load and the control load. The higher the frequency is, the greater the velocity and loads are. The communications load is greater because the sampling data has to be sent to logical sensors, the control load is greater because there is more data to process and the navigation velocity can be greater because the robot can avoid obstacles or to guide the course quicker.

**Figure 28:**
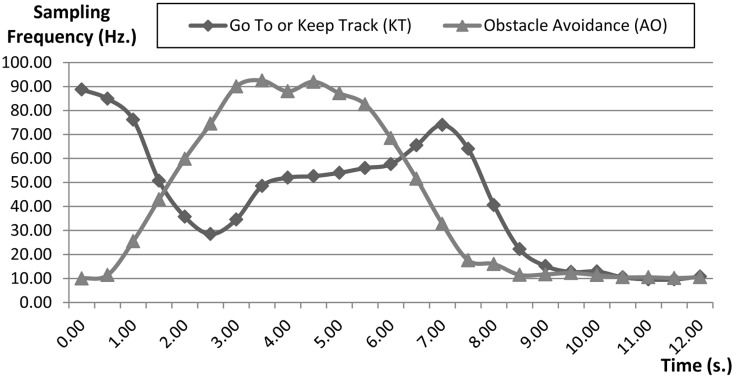
Sampling rates of “obstacle avoidance (OA)” and “go to o keep track (KT)” behaviours during navigation without optimization (Path I).

The FSACtrl architecture allows the system to dynamically change the sampling rate in order to adapt this frequency in the context. For example, when the robot detects an obstacle the system could decide to increase the sampling rate to react more quickly. The aim is to optimize the communications and control load without impairing the navigation speed. In the first optimization, the system tries to move the robot the quickest possible with the minimum communications load. Figure 28 shows, besides, the average values of the velocity and loads when the vehicle is moved by using dynamic sampling periods. The average value of the speed of the robot is better than expected based on the average value of the sampling rate. In Figure 28 sampling rates of “obstacle avoidance (AO)” and “go to (KT)” behaviours, which have been used during the navigation, are shown.

Initially, the sampling rate of the “obstacle avoidance” (AO) behaviour is low because the distance sensors detect that the obstacles are very far away. When the wall is detected the sampling rate is increased to let the robot turn more quickly. The time when the sampling rate is greater is when the robot turns the first corner, between times 3 and 4, and the second corner, between times 4 and 5. After that, the sampling rate of the “obstacle avoidance” decreases gradually because there are no more obstacles.

In the case of the “go to” behaviour (KT), initially the sampling rate is high because the robot is not oriented and has to navigate the course. Once the robot can be moved towards the destination (without drift) the sampling rate decreases. However, when the vehicle avoids an obstacle the robot has to change its course and then the control error of the “go to” behaviour increases. As a result, the sampling rate of the “go to” behaviour increases, too.

**Figure 29:**
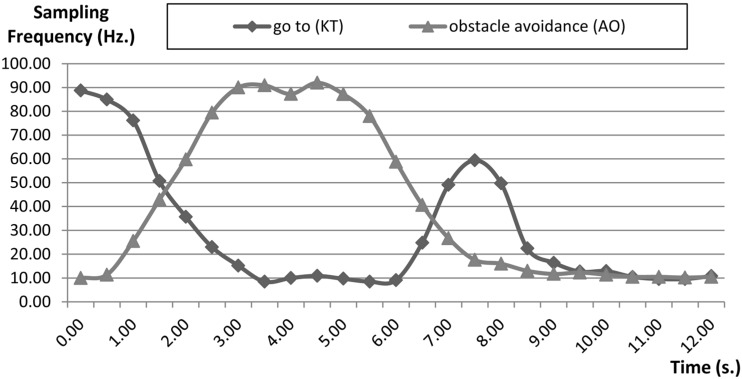
Optimisation of the sampling rate of “go to or keep track (KT)” behaviour during navigation (Path II).

However, this increase of the sampling rate of the “go to” behaviour does not imply one gets a better course because of the “obstacle avoidance” behaviour has more priority than the “go to” behaviour, so a second optimization is to decrease the sampling rate of the “go to” behaviour while the robot is avoiding an obstacle. This optimization reduces the control load because there is less processing. Results are shown in Figure 29.

**Figure 30:**
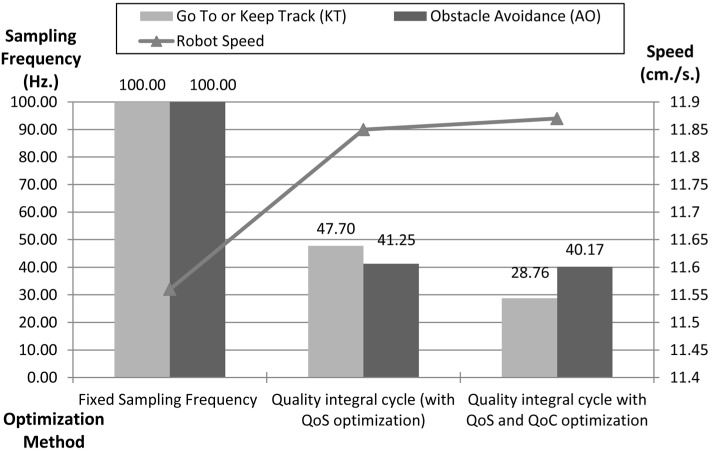
Optimization obtained by the dynamic management of communications and control.

Figure 30 shows a comparison of the tests and optimizations. The results validate how the dynamic management of communications and control reduces the network and control load while maintaining the navigation performance.

To compare the methods, the higher sampling frequency (100 Hz) it has been chosen as the basic method because this frequency provides the fastest robot navigation speed in the proposed scenario and allows one to compare the results as a percentage of this value. This speed is obtained by matching the dynamic management of the sampling frequency for both optimization methods employed.

When the system uses the quality integral cycle, using only QoS to optimize the control component, the average sampling frequency obtained is 47.7 Hz for the Keep Track behaviour and 41.25 Hz for the Obstacle avoidance behaviour. When system uses the quality integral cycle, using both QoS and QoC optimization, if the obstacle avoidance behaviour works then the Keep Track behaviour is stopped due to the fact that obstacle avoidance is the highest priority. As a result, the navigation speed is similar and however the sampling frequency of the “keep track” is significantly reduced. Consequently the control component load is optimized due to the use of the quality integral cycle.

## 6. Conclusions

This paper has presented an architecture based on standards that can determine system characteristics, evaluate system performance using parameters, and use QoS policies to optimize performance relative to the control characteristics.

The presented architecture (FSACtrl) has several interesting features: it is modular and scalable because it organizes the basic control elements into a well-known hierarchy. It supports the DDS communications model by OMG, allowing implementation of open systems. It also provides an interesting support to the measurement of QoS and QoC using simple parameters that have been validated in experiments.

The architecture presented proposes a model for event management, called “quality integral cycle”, which gives support to the control mechanism. With the quality integral cycle the system can self-tune to optimize the load of the control elements.

The architecture has been validated by means of a series of tests based on Braitenberg vehicles. Tests involved measures of how QoS and QoC parameters can monitor the use of system resources and can be used to optimize the navigation of a mobile robot. The reactive navigation has been tested using the 1, 2, 3 and 4 Braitenberg vehicles and the behaviour-based navigations has been tested using the Braitenberg 5 vehicle.

Regarding the theoretical contributions, the present work presents a way to measure with specific parameters the QoC and QoS in mobile robots. The proposed formulas of QoC are adapted for the specific reactive (sensor-based) behaviour: go to. The proposed formulas of QoS can be used in every distributed system and allow the client to adjust the sensor behaviour, for example changing the acquisition process parameters (like the sampling period). The formulas are used to measure the optimization, in terms of control and communications, of the robot navigation. The experiments carried out and presented in this paper demonstrate the convenience of using the QoS and QoC working together in distributed sensor-based control systems.

As future work, several research lines can be studied. The use of DDS middleware-based communications is very interesting, and to study how to implement the middleware in the sensors and actuators of the system is one of the research lines that is currently being worked on [38].

Another line of interest is to extend the definition of QoS and QoC parameters. It is possible to use QoS policies based on DDS to define complex parameters to measure the QoC of the service. Besides, defining QoC parameters based on the specific characteristics of mobile robot navigation behaviours enables control actions to be closer to these navigation behaviours, and therefore to optimize these behaviours. The article presents how to use QoS and QoC parameters with two robot navigation behaviours: “go to a point” and “obstacle avoidance”. However, it would be interesting to extend the QoC parameters to other robot navigation behaviours, such as monitoring of walls, door steps or avoidance of dynamic obstacles.
